# Outlines of the Nerves, with Short Descriptions

**Published:** 1846-02

**Authors:** 


*Outlines of the Nerves, with short descriptions. Designed for the use of Medical Students.* By John Neill, M. D., Demonstrator of Anatomy in the University of Pennsylvania; Physician to Wills' Hospital; Lecturer on Anatomy, etc. etc. etc. Philadelphia: Ed. Barrington & Geo. D. Haswell. 1845.

Our favorable anticipations have been more than realized in the appearance of this accompaniment to "Outlines of the Arteries," by the same author, which has already become so favorably known. The great and *distinctive* merit in that work, of placing the name upon the vessels and their branches, has also been observed in this; and the student is enabled to see, at a single glance, both the main trunk and the filaments arising from it. This advantage is particularly conspicuous in plate 3d, where the fifth pair and its various branches are beautifully, and, what is better, *clearly* displayed. The difficulty of following a demonstration, so often complained of by students, can, we think, no longer exist with such a guide as this.

The author does not pretend to portray all the minutiæ of the discoveries of the nervous system; his great aim is to render clear what is already known, and to give to students an easy method of acquiring it. To accomplish this, the plan of demonstration already mentioned has been adopted, and the plates have been altered from others, so as to suit the terms and descriptions of the standard works of the day, thus rendering it a suitable accompaniment to almost any text book.

The drawings are faithfully and beautifully done, and we feel sure that it will obtain a large share of the approbation it so deservedly merits. We understand that a similar work, on the Veins and Lymphatics, by the same author, will shortly appear, which will render the series complete. The present work is neatly and substantially gotten up, and reflects great credit on all concerned. F. G. S.

